# Transport starter data kit: Historical passenger and freight transport data for selected countries in Africa, Asia, and South America

**DOI:** 10.1016/j.dib.2024.110984

**Published:** 2024-10-09

**Authors:** Naomi Tan, Robert Ambunda, Nikola Medimorec, Angel Cortez, Agustina Krapp, Erin Maxwell, John Harrison, Mark Howells

**Affiliations:** a Centre for Sustainable Transitions: Energy, Environment and Resilience, Loughborough University, United Kingdom; b Centre for Environmental Policy, Imperial College London, United Kingdom; c SLOCAT Partnership on Sustainable, Low Carbon Transport, Belgium

**Keywords:** Transport, Transport systems, MAED, OSeMOSYS

## Abstract

The demand for data-driven models to inform sustainable transportation planning has become more important as countries address the complexities of urban mobility. However, data collection and curation are time-consuming and can be challenging due to data inaccessibility and inaccuracy. The Transport Starter Data Kit therefore aims to address these challenges, offering a one-stop-shop for transport modelling-related data. The Kit contains historical annual data (1990–2021) on passenger and freight activity, energy intensities, load factors, and vehicle stock, segregated by mode and fuel where available. Additionally, population and GDP data, which influence transport activity, are included. The value of the dataset lies not only in the range of variables it offers but also in the compilation from multiple authoritative sources, providing researchers, consultants, and policymakers interested in data-based transport modelling with a foundational base for their model development. By adopting, adapting, and applying the data, clear policies may be developed which can underpin the necessary finances for sustainable transport development.

Specifications Table

**Table:** 

Subject	Transportation Management
Specific subject area	Transport Systems Modelling
Data format	Raw, Analyzed
Type of data	Table, Graph
Data collection	Historical data from 1990 to 2021 were collected from the websites, annual reports, and databases of international organisations, as well as from academic articles and existing modelling databases. The data were collected for use with the Model for Analysis of Energy Demand (MAED) tool, which can project transport demand based on historical data. Nonetheless, the data available through this document is independent of the tool.
Data source location	Raw data sources are listed in the references section and Table 1 of this article.
Data accessibility	Repository name: Zenodo Data identification number: 10.5281/zenodo.8060153 Direct URL to data: https://zenodo.org/records/8060153

## 1 Value of the Data

• The socio-transport data are useful for country analysts, policymakers, and the broader scientific community, to understand historical transport trends or as a base for systems model development for specific countries to inform national transport investment outlooks and policy plans. For example, by analyzing trends in passenger transport demand using this dataset, policymakers can identify patterns that may influence future infrastructure investments or environmental policies.
• The data is compiled in one repository, which reduces the data collection time, thus, can be useful for capacity building events with limited time. To date, the dataset has been used in Climate Compatible Growth's Energy Modelling Platforms, as well as Master's theses at Imperial College London and University College London to investigate forecasted transport demand and develop capacity expansion plans for electrification of the transport sector [1].
• The data can be used with the tools Model for Analysis of Energy Demand (MAED) and the Open Source Energy Modelling System (OSeMOSYS) to project future transport demand and installed capacity needed for the required demand, respectively. This promotes the use of evidence-based decision-making for the transport sector, which is increasingly important as countries seek to develop sustainable and efficient transport systems alongside the power sector. Nonetheless, the data collected in this paper are independent to these tools.
• The data are open-source and country-specific which is not easily accessible in current literature. By compiling secondary data from multiple, diverse sources, the work provides analysts with complete and accessible datasets, helping to overcome barriers of data inaccessibility.
• The data not only provides a range of transport and macroeconomic variables but also offers data compiled from multiple authoritative sources for added reliability.
• The dataset promotes the U4RIA goals [2], which are Ubuntu, Retrievability, Reusability, Repeatability, Reconstructability, Interoperability, and Auditability.

## 2 Data Description

This paper presents historical socio-transport data from 1990 to 2021 by country and related data by region within selected countries in Africa, Asia, and South America. The selected list of countries can be found in Table 5. The time period and countries are selected based on relevancy and data availability, and their data can be used as input to develop a transport system model for the selected countries. The data collected were from publicly available sources, including the reports of international organizations, journal articles, and existing databases, which complies with the U4RIA goals, which stand for Ubuntu, Retrievability, Reusability, Repeatability, Reconstructability, Interoperability, and Auditability. In more detail, the U4RIA goals are designed to improve transport modelling for policy and financial support through guidelines and best practices [2]. The methods of data collection and preparation are described in Section 2 of this article. The data sources used are listed in Table 1, with each source given a reference code which will be referred to throughout the paper. The dataset includes raw data on population, gross domestic product (GDP), as well as passenger activity, freight activity, vehicle stock number, energy efficiency, and load factor by mode, where available. Social data on population and GDP are included as transport demand are dependent on both factors [3]. GDP share by sector (agriculture, construction, mining, manufacturing, services, and energy) were processed based on assumptions. This was produced to comply with the Model for Analysis of Energy Demand (MAED) tool [4], nonetheless, the data provided in this paper are independent to the tool. For transparency and easy uptake, each data is also given an observation code based on the Statistical Data and Metadata eXchange (SDMX) Code List for Observation Status, which can be found in the dataset file. For further comprehensive understanding, country-specific datasets are available externally for each country (see Table 5 within the Appendix for links to each available country-specific dataset). Most data in the Transport Starter Data Kit were collected from The World Bank (TWB) DataBank (DB) [5], United Nations (UN) Department of Economic and Social Affairs (DESA) [6,7], International Road Federation (IRF) World Road Statistics (WRS) [8], International Union of Railways (UIC) Statistics Rail Information System and Analyses (RAILISA) [9], Asian Development Bank (ADB) Asian Transport Outlook (ATO) [10], and the Statistical, Economic and Social Research and Training Centre for Islamic Countries (SESRIC) Statistical Yearbook [11]. Further data sources used are listed in Table 1.

**Table 1:** Additional data sources used in the paper, segregated by country and continent.

Continent	Country	References
Africa	Algeria	Algeria Data Portal (2023). Dataset Browser. Available at: https://algeria.opendataforafrica.org/data/#menu=topic
	Angola	OICA (2022). Vehicles in use. Available at: www.oica.net/category/vehicles-in-use/
		Gwilliam, K. (2011). Africa's Transport Infrastructure, Mainstreaming Maintenance and Management, World Bank. Available at: https://ppiaf.org/documents/3136/download
	Benin	Direction de la Programmation et de la Prospective (2014). Annuaire Statistique des Transports 2001–2008. Available at: https://transports.bj/wp-content/uploads/2018/03/Annuaire_Statistique_TPT_2001_2008_VF.pdf; Ministere du Plan et du Developpement (2019); Tableau de Bord Social (2015). Available at: https://instad.bj/images/docs/insae-statistiques/sociales/Tableau%20de%20Bord%20Social/Tableau%20de%20Bord%20Social%202015.pdf; Ministere des Infrastructures et des Transports (2017). Annuire Statistique 2013–2016. Available at: https://transports.bj/wp-content/uploads/2018/03/Annuaire_Statistique_TPT_2013_2016_VF.pdf; Institut National de la Statistique et de l'Analyse Economique (2019). Annuaire Statistique 2019. Available at: https://instad.bj/images/docs/insae-publications/annuelles/AS-INSAE/Annee_2019/Annuaire_Statistique_National_2019.pdf
	Botswana	Statistics Botswana (2021). Transport and Infrastructure Statistics Report. Available at: https://www.statsbots.org.bw/sites/default/files/publications/2020%20Transport%20and%20Infrastrucutre%20Statistics%20Report.pdf
	Burkina Faso	SSATP (2019). Policies for sustainable mobility and accessibility in cities of Burkina Faso. Available at: https://www.ssatp.org/sites/ssatp/files/publication/Country-Assesment-report-Burkina%20Faso-En.pdf
	Cameroon	National Institute of Statistics of Cameroon (2014). Cameroon Statistical Yearbook 2014. Available at: https://cameroon.opendataforafrica.org/ggtphlc;
		Ministry of Transport (2019). Transport Statistics Yearbook 2019 Edition. Available at: http://mintransports.net/Annuaire-Statisrique-du-MINT_Version_Anglaise_OK.pdf
	Côte d'Ivoire	Institut National de la Statistique (2012). Annuaire des Statistiques Demographiques et Sociales. Available at: http://www.ins.ci/templates/Pub/annuaire%20demo.pdf
	Djibouti	Djibouti Data Portal (2022), Transports. Available at: https://djibouti.opendataforafrica.org/acqjnyc/transports
	Egypt	Knoema (2020). Egypt - Air transport freight. Available at: https://knoema.com/atlas/Egypt/Air-transport-freight
		Egypt Data Portal (2023). Dataset Browser. Available at: https://egypt.opendataforafrica.org/data/#topic=Transport
	Equatorial Guinea	Equatorial Guinea Data Portal (2023). Dataset Browser. Available at: https://eguinea.opendataforafrica.org/data/#topic=Transport
	Eswatini	Eswatini Data Portal (2023). Dataset Browser. Available at: https://swaziland.opendataforafrica.org/data/#topic=Transport
	Ethiopia	The Federal Democratic Republic of Ethiopia (2020). Transport Sector - Ten Years Perspective Plan (2020/21 - 2029/30). Available at: http://www.motr.gov.et/web/guest/-/ministry-of-transport-10-year-pl-2?inheritRedirect=true
		Desta, D. (2021). Assessment of after-sales service management in the case of Motor and Engineering Company of Ethiopia (MOENCO). Available at: http://213.55.95.56/bitstream/handle/123456789/27454/Dawit%20Desta.pdf?sequence=1&isAllowed=y
		Addis Ababa Institute of Technology (2012). Final report on pilot Global Fuel Economy Initiative study in Ethiopia. Available at: https://www.globalfueleconomy.org/in-country/africa
		TricksFast (2020). Ethiopia registered vehicle reaches 1.2 million. Available at: https://tricksfast.com/ethiopia/ethiopia-registered-vehicle-reaches-1-2-million/
	Gabon	Gabon Data Portal (2023). Dataset Browser. Available at: https://gabon.opendataforafrica.org/data/#menu=topic
	Gambia	Gambia Data Portal (2023). Dataset Browser. Available at: https://gambia.opendataforafrica.org/data/#menu=topic
	Ghana	Lee, N. (2016). Decision support methodology for national energy planning in developing countries: an implementation focused approach. Available at: https://www.semanticscholar.org/paper/Decision-support-methodology-for-national-energy-in-Lee/7383d3289f197b180cf7b1cdd7222570f358f4eb
		Ghana Statistical Service (2015). Ghana's Statistical Year Book 2010–2013. Available at: https://statsghana.gov.gh/gsspublications.php?category=MTAyOTI2NTM5NC42ODM1/webstats/8no4s4pq66
		Table B-1 - Calculated values for a study, references mentioned are Anin, E.K., Annan, J., Otchere, A.F. (2013). Evaluating the role of mass transit and its effect on fuel efficiency in the Kumasi metropolis, Ghana. Int. J. Bus. Soc. Res. 3, 107–116; UITP, UATP (2010). Report on statistical indicators of public transport performance in Africa. Union Internationale des Transports Publics (UITP & Union Africaine des Transports Publics (UATP), Brussels. Available at: https://www.researchgate.net/publication/358107686_Evaluating_the_Role_of_Mass_Transit_and_its_Effect_on_Fuel_Efficiency_in_the_Kumasi_Metropolis_Ghana
	Kenya	JICA (2006). The Study on Master Plan for Urban Transport in the Nairobi Metropolitan Area. Available at: https://openjicareport.jica.go.jp/pdf/11823093_03.pdf
		KNBS (2021). Statistical Abstract 2021. Available at: https://www.knbs.or.ke/download/statistical-abstract-2021/
		GIZ TraCS (2018). Greenhouse gas emissions from the transport sector: Mitigation options for Kenya. Available at: https://www.changing-transport.org/wp-content/uploads/2018_GIZ_INFRAS_Transport_Mitigation_Options_Kenya.pdf
	Malawi	Malawi (2023). Dataset Browser. Available at: https://malawi.opendataforafrica.org/data/#menu=topic
	Mali	Mali Data Portal (2023). Dataset Browser. Available at: https://mali.opendataforafrica.org/data/#menu=topic
	Mauritania	Mauritania Data Portal (2023). Dataset Browser. Available at: https://mauritania.opendataforafrica.org/data/#menu=topic
	Morocco	Morocco Data Portal (2023). Dataset Browser. Available at: https://morocco.opendataforafrica.org/data/#menu=topic
	Mozambique	Instituto Nacional de Estadística (2020). Estadísticos dos transportes e comunicações. Available at: http://www.ine.gov.mz/estatisticas/estatisticas-sectoriais/transporte-e-comunicacao
	Nigeria	Dioha, M. and Kumar, A. (2020), Sustainable energy pathways for land transport in Nigeria, Utilities Policy, Volume 64, 101034, ISSN 0957-1787, https://doi.org/10.1016/j.jup.2020.101034. Available at: https://www.sciencedirect.com/science/article/abs/pii/S0957178720300291?via%3Dihub
		Federal Republic of Nigeria (2020). Third National Communication of the Federal Republic of Nigeria. Available at: https://unfccc.int/sites/default/files/resource/NIGERIA_NC3_18Apr2020_FINAL.pdf
	Senegal	Agence Nationale de la Statistique et de la Demographie (2020). Situation Economique et Sociale du Sénégal Ed. 2017/2018. Available at: https://www.ansd.sn/sites/default/files/2023-03/13-SES-2017-2018_Transport.pdf
	South Africa	Havenga, J.H., Simpson, Z.P., King, D. de Bod, A. and Braun, M. (2016). Logistics Barometer South Africa 2016. Stellenbosch University. Available at: http://www.sun.ac.za/english/faculty/economy/logistics/Pages/logisticsbarometer.aspx
		Venter, C. and Mohammed, S.O. (2013). Estimating car ownership and transport energy consumption: A disaggregate study in Nelson Mandela Bay. Journal of the South African Institution of Civil Engineering. 55. 2–10. Available at: https://www.researchgate.net/publication/282543048_Estimating_car_ownership_and_transport_energy_consumption_A_disaggregate_study_in_Nelson_Mandela_Bay
	Tunisia	Statistiques Tunisie (2023). Tunisia Database. Available at: http://dataportal.ins.tn/en
	Uganda	Uganda Data Portal (2023). Dataset Browser. Available at: https://uganda.opendataforafrica.org/data/#topic=Transport
	United Republic of Tanzania	National Bureau of Statistics (2019). Tanzania Socio-Economic Database. Available at: http://www.tsed.go.tz/
		African Development Bank (2013). Tanzania Transport Sector Review. Available at: https://www.afdb.org/fileadmin/uploads/afdb/Documents/Project-and-Operations/Tanzania_-_Transport_Sector_Review.pdf
	Zambia	Zambia Ministry of Transport and Communication (2017). MTC Sector Performance. Available at: https://zambiamtc.opendataforafrica.org/kenvome/mtc-sector-performance; Ministry of Transport and Communications (2019). 2019 Annual Report. Available at: https://www.motl.gov.zm/?page_id=1260#1635868305817-8b615f21-d43c
	Zimbabwe	National Statistics Agency (2018). Zimbabwe Statistics 2010. Available at: https://zimbabwe.opendataforafrica.org/awadaad/zimbabwe-statistics-2010
	Taiwan Province of China	United Nations, Department of Economic and Social Affairs, Population Division (2022). World Population Prospects 2022. Online Edition. Available at: https://population.un.org/wpp/
		International Monetary Fund (2023). World Economic Outlook. Available at: https://www.imf.org/en/Data
		Taiwan National Statistics (2023). Breakdown of gross domestic product (GDP) of Taiwan from 2012 to 2022, by economic sector. Available at: https://www.statista.com/statistics/321366/taiwan-gdp-breakdown-by-sector/
	Viet Nam	General Statistics Office of Vietnam (2020). Available at: https://www.gso.gov.vn/en/homepage/
South America	Brazil	Goes, G. V., Gonçalves, D. N. S., de Almeida D'Agosto, M., La Rovere, E. L., & de Mello Bandeira, R. A. (2020). MRV framework and prospective scenarios to monitor and ratchet up Brazilian transport mitigation targets, Climatic Change, doi:10.1007/s10584-020-02767-6. Available at: https://link.springer.com/article/10.1007/s10584-020-02767-6

### 2.1 Population

**Fig. 1:**
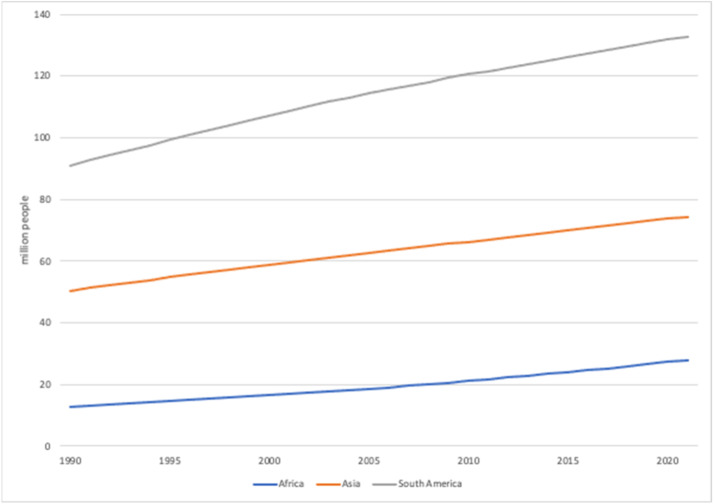
Average population (million people) of Africa, Asia, and South America. Note that only 10 countries in Asia and 2 countries in South America, following the countries selected in the dataset, were used to calculate the average. Thus, these averages are not representative of the entire continent.

Annual population count (million people), population share by urban and rural (%), and population growth from 1990 to 2021 (%) for the selected countries are presented in the dataset. Data was directly collected from the World Bank DataBank [5]. Using the dataset, various trends can be analyzed. For example, the average population for each continent can be analyzed (Fig. 1).

### 2.2 Gross domestic product (GDP)

**Fig. 2:**
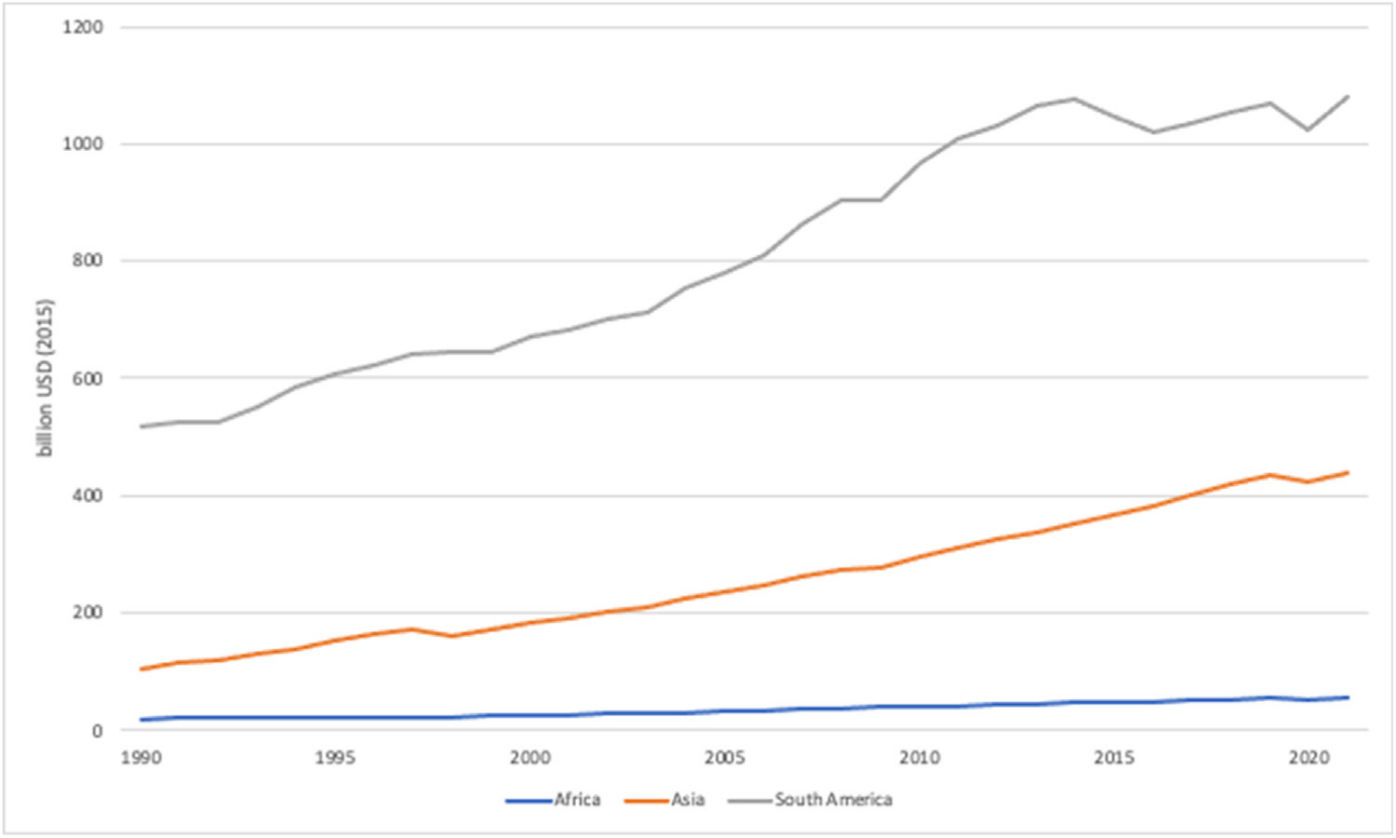
Average GDP (billion USD (2015)) of Africa, Asia, and South America. Note that only 10 countries in Asia and 2 countries in South America, following the countries selected in the dataset, were used to calculate the average. Thus, these averages are not representative of the entire continent.

**Fig. 3:**
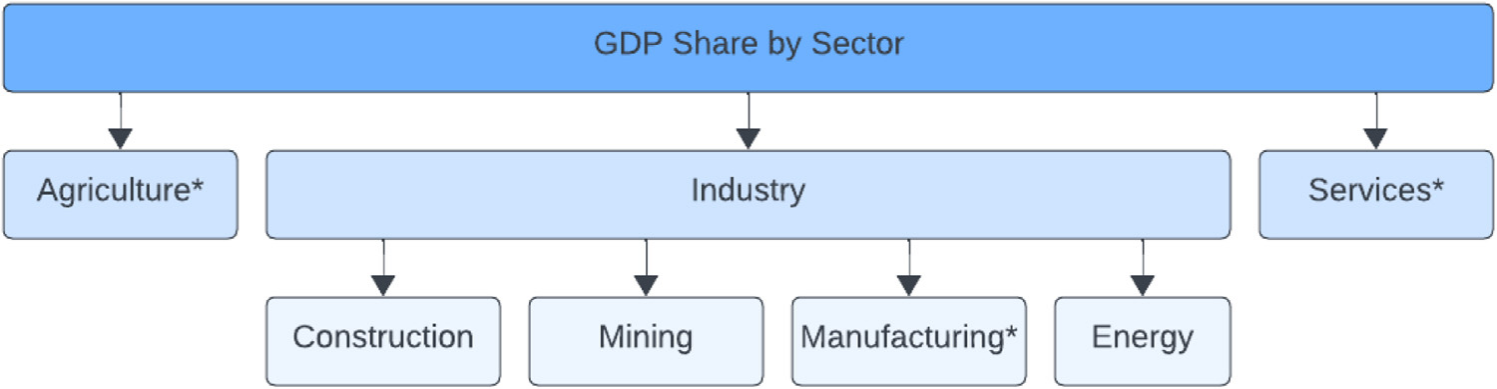
Simplified diagram of the different sectors that contribute to overall GDP. An asterisk (*) denotes that the data for the sector is provided by The World Bank DataBank [5].

Annual GDP (million USD (2015)), GDP by sectorial share (agriculture, construction, mining, manufacturing, services, and energy) (%), and GDP growth (%) from 1990 to 2021 for the selected countries are presented in this paper. Annual GDP and GDP growth are collected directly from the World Bank DataBank [5]. Where data was not available, data processing was done. Nonetheless, trends for each continent can be analyzed (Fig. 2). GDP share by sector was also further processed, with both the methodologies outlined in Section 2 (Fig. 3).

### 2.3 Passenger activity

Passenger activity (million passenger-km) for road, rail, air, and inland waterways are noted in the dataset for all selected countries. Passenger activity by mode is also displayed, where available. These data were mainly collected from TWB DB [5], UN DESA [6], IRF WRS [8], the UIC RAILISA [9], and the ADB ATO [10]. Country-specific databases and articles from which data were collected from are listed in Table 1. These data were collected directly from the sources.

### 2.4 Freight activity

Freight activity (million ton-km) for road, rail, air, and inland waterways from the year 2015 onwards are noted in the dataset for all selected countries. These data were mainly collected from TWB DB [5], UN DESA [7], IRF WRS [8], UIC RAILISA [9], ADB ATO [10], and the SESRIC Statistical Yearbook [11]. Country-specific databases and articles from which data were collected are listed in Table 1. These data were collected directly from the sources. T

### 2.5 Vehicle stock number

Vehicle stock number (units) for various road transport modes (motorcycle, car, bus, light-duty vehicle, heavy-duty vehicle, minibus, agricultural and forestry tractors, special purpose vehicles, and so on) are listed in the dataset for all selected countries.. These data were mainly collected from the IRF WRS [8] and the ADB ATO [10]. Country-specific databases and articles from which data were collected are listed in Table 1. Most of the data were collected directly from the sources, with a small number calculated with the methodology described in Section 2.

### 2.6 Energy intensity

Energy efficiency data in the unit of MJ/passenger for different vehicle and fuel types, where available, in Africa, Asia, and South America were collected directly from Kane [12], International Energy Agency (IEA) [13], and Goes [14], respectively (Table 2). Due to lack of available data, it was assumed that energy efficiency within each continent were the same. Energy efficiency data for megajoules/vehicle-kilometre was also obtained from Venter and Mohammed [15], however this was location-specific to South Africa.

**Table 2:** Energy efficiency (megajoules/passenger) of different vehicle and fuel types, where available, in Africa, Asia, and South America.

Region	Year	Vehicle mode	Energy efficiency	Unit
Africa	2013	Car (electric)	0.55	MJ/passenger
		Car (hybrid)	1.56	MJ/passenger
		Car (petrol)	2.22	MJ/passenger
		Minibus (petrol)	0.66	MJ/passenger
Asia	2018	Motorcycle	0.5	MJ/passenger
		Car	1.8	MJ/passenger
		Minibus	0.7	MJ/passenger
		Bus	0.7	MJ/passenger
		Light-duty vehicle	2.7	MJ/passenger
		Rail	0.2	MJ/passenger
		Aviation	1.8	MJ/passenger
South America	2005, 2010, 2015, 2017	Car	1.1	MJ/passenger
**Country**	**Year**	**Vehicle mode**	**Energy efficiency**	**Unit**
South Africa	2004	Motorcycle	102.8	MJ/vehicle-km
		Car	396.4	MJ/vehicle-km
		Minibus	513.8	MJ/vehicle-km
		Bus	1833.5	MJ/vehicle-km
		Light-duty vehicle	451.4	MJ/vehicle-km
		Rail (passenger)	10.3	MJ/vehicle-km

### 2.7 Load factor

Load factors (passenger/vehicle) for different vehicle and fuel types, where available, for Africa and South America are noted in Table 3. Due to lack of available data, the load factor from Kane [12] is assumed to be representative of Africa. Similarly, the load factor from Oviedo [16] is assumed to be representative of South America. Data for Asia was unavailable. Nonetheless, country-specific data for ten African countries: Côte d'Ivoire, Cameroon, Ethiopia, Ghana, Kenya, Namibia, Nigeria, Senegal, United Republic of Tanzania, and South Africa, are available directly from Merven [17].

**Table 3:** Load factors (passengers per vehicle) for different vehicle and fuel types, where available, in Africa and South America. Country-specific load factors for ten African countries are also noted in the table.

Region	Year	Vehicle mode	Load factor
Africa	2013	Car (electric)	1.4
		Car (hybrid)	1.4
		Car (petrol)	1.4
		Minibus (petrol)	7.8
South America	2018	Car	1.2
		Bus	36
**Country**	**Year**	**Vehicle mode**	**Load factor**
Côte d'Ivoire	2010	Car	2
		Minibus	18
		Bus (diesel)	60
Cameroon	2010	Car	2.3
		Minibus	17
		Bus (diesel)	45
Ethiopia	2010	Car	3.7
		Minibus	11
		Bus (diesel)	80
Ghana	2010	Car	2
		Minibus	18
Kenya	2010	Car	1.7
		Minibus	18
		Bus (diesel)	70
Namibia	2010	Car	1.3
Nigeria	2010	Car	1.8
		Minibus	18
		Bus (diesel)	43
Senegal	2010	Car	2
		Minibus	35
		Bus (diesel)	66
United Republic of Tanzania	2010	Car	1.9
		Minibus	29
		Bus (diesel)	45
South Africa	2010	Car	1.4
		Minibus	8.5
		Bus (diesel)	37.1

## 3 Experimental Design, Materials and Methods

Data were primarily collected from the databases and websites of international organizations, including TWB DB [5], UN DESA [6,7], IRF WRS [8], UIC RAILISA [9], ADB ATO [10], SESRIC Statistical Yearbook [11], and the IEA [13]. Additionally, data were sourced from existing studies, country reports, and national websites from researchers, organizations, and government as noted in Table 1. The subsections below note how the data were collected and processed in more detail.

### 3.1 Population

Data for population including total population, urban population, and population growth are from TWB DB [5]. Only one country, Taiwan Province of China, had missing urban population share data for various years, to which the authors addressed by extrapolating from the available historical years. This was done by calculating the difference in urban population share percentage between years with available data. The difference was then divided by the number of years with missing values. This method allowed for a fast and consistent approach to addressing missing values. However, it is acknowledged that this may not fully capture the abrupt changes in urbanization patterns. Nonetheless, it provides a complete dataset for population analyses.

### 3.2 GDP

Data for GDP including total GDP, GDP share by sector, and GDP growth are from TWB DB [5]. Similar to population, several countries had missing data for certain years. In this case, the approach that was done with population was repeated, where data was extrapolated from the available historical years. Where data were missing at the start of the investigation period only, the average increase or decrease rate was calculated based on the next 15 years, and applied to the missing values. Likewise, where data were missing at the end of the investigation period only, the average increase or decrease rate was calculated based on the former 15 years, and applied to the missing values. In the instance where there were not an adequate number of years to extrapolate, or where the extrapolation led to unrealistic numbers, an overall average was calculated based on the available data. Table 4 notes the countries where data was extrapolated or averaged.

**Table 4:** List of countries and their data processing methodology. An asterisk (*) denotes that he country had both extrapolation and averaging as the methodology.

None		Extrapolation		Average
Egypt	United Republic of Tanzania	Algeria	Liberia*	Djibouti
Eswatini	Togo	Angola	Libya	Equatorial Guinea
Ethiopia	Tunisia	Benin	Malawi	Liberia*
Gabon	Uganda	Botswana	Mali	South Sudan*
Ghana	Zambia	Burkina Faso	Mauritania	Myanmar*
Morocco	Indonesia	Burundi	Mozambique	
Namibia	Lao People's Democratic Republic	Cameroon	Senegal	
Niger	Malaysia	Central African Republic	Sierra Leone	
Nigeria	Republic of Korea	Chad	Somalia	
Rwanda	Brazil	Congo	South Sudan*	
South Africa	Colombia	Côte d'Ivoire	Sudan	
		Democratic Republic of the Congo	Zimbabwe	
		Eritrea	Cambodia	
		Gambia	Myanmar*	
		Guinea	Philippines	
		Guinea-Bissau	Taiwan Province of China	
		Kenya	Thailand	
		Lesotho	Viet Nam	

Further data processing was done with all countries to obtain GDP share by sector. TWB DB provided GDP share by sector for agriculture, manufacturing, and services. However, GDP share by construction, mining, and energy was also needed to align the data structure with the MAED tool. To address the lack of data available for these sectors, the authors assumed that construction, mining, manufacturing, and energy all fall within the industry sector. Thus, to obtain data for the three remaining sectors, the remaining percentage after considering agriculture, manufacturing, and services from TWB DB [5], was divided by three. It is therefore assumed that the GDP share of the construction, mining, and energy sectors are the same. Fig. 3 shows an illustrative diagram of the methodology.

### 3.3 Transport activity and vehicle stock number

The disaggregation of vehicle modes for passenger activity, freight activity, and vehicle stock number follows the European Union vehicle classification, which is based on the United Nations Economic Commission for Europe (UNECE) standards [18]. It should be noted that none of the passenger and freight activity were processed. Data processing was only done to a few of the countries’ vehicle stock number. Whereby annual vehicle registration number was found instead of annual vehicle stock number, annual (new) vehicle registration number was added to give a cumulative total each year, representing vehicle stock number. This methodology was done for Kenya, Mozambique, Uganda, Zambia, and Colombia. Further, where total road freight data was only found, this was split into two for some countries to cover light-duty vehicles and heavy-duty vehicles, assuming that the two will be the same. This was done for the Democratic Republic of the Congo and Egypt.

### 3.4 Energy intensity

The disaggregation of vehicle modes for passenger activity, freight activity, and vehicle stock number follows the European Union vehicle classification, which is based on the United Nations Economic Commission for Europe (UNECE) standards [18]. Due to lack of available country-specific data, energy intensity levels as calculated for South Africa [12] and Brazil [14] are assumed to be representative for the rest of Africa and South America, respectively. Similarly, global average values as calculated by the IEA [13] are assumed to represent the Asia region.

### 3.5 Load factor

The disaggregation of vehicle modes for passenger activity, freight activity, and vehicle stock number follows the European Union vehicle classification, which is based on the United Nations Economic Commission for Europe (UNECE) standards [18]. Due to lack of available country-specific data, load factor data as calculated for South Africa [12] and Colombia [16] are assumed to be representative for the rest of Africa and South America, respectively.

## Limitations

As shown in Table 2, Table 3, and the dataset, available open-access transport data is challenging to find. Thus, there are several years with empty cells, due to countries or international organizations not monitoring or publishing the related country statistics. Further, some data may be deprecated, such as several data from UN DESA [6,7]. This paper publishes these data regardless due to lack of readily available data. There are also a few discrepancies with certain values from different sources. For example, there is a sudden large increase from 2018 to 2019 for Cameroon's passenger activity. Numerous assumptions were also made to the population, GDP, and transport data, as stated in Section 2, to address data gaps. Thus, these data may lack a high level of reliability. Nonetheless, the data presented can be used as a foundational base for transport demand modelling using MAED, carbon modelling using OSeMOSYS, or others, for the 60 different countries in Africa, Asia, and South America listed in Table 5.

## Ethics Statement

The authors have read and followed the ethical requirements for publication in Data in Brief and confirm that the current work does not involve human subjects, animal experiments, or any data collected from social media platforms.

## CRediT Author Statement

**Naomi Tan:** Conceptualization, Methodology, Data curation, Writing – Original draft preparation, Validation, Visualization. **Robert Ambunda:** Data curation, Validation. **Nikola Medimorec:** Data curation, Validation. **Angel Cortez:** Data curation, Validation. **Agustina Krapp:** Data curation, Validation. **Erin Maxwell:** Data curation, Validation. **John Harrison:** Supervision, Writing – Reviewing and Editing. **Mark Howells:** Supervision.

## Figures and Tables

**Table 5:** List of links to the country-specific Transport Starter Data Kit dataset and data note.

Continent	Country	Link
Africa	Algeria	https://doi.org/10.5281/zenodo.7997446
	Angola	https://doi.org/10.5281/zenodo.6539080
	Benin	https://doi.org/10.5281/zenodo.6539105
	Botswana	https://doi.org/10.5281/zenodo.6539124
	Burkina Faso	https://doi.org/10.5281/zenodo.7998168
	Burundi	https://doi.org/10.5281/zenodo.7998154
	Cameroon	https://doi.org/10.5281/zenodo.6539144
	Central African Republic	https://doi.org/10.5281/zenodo.7997531
	Chad	https://doi.org/10.5281/zenodo.7998141
	Congo	https://doi.org/10.5281/zenodo.7998237
	Côte d'Ivoire	https://doi.org/10.5281/zenodo.6539150
	Democratic Republic of the Congo	https://doi.org/10.5281/zenodo.7998129
	Djibouti	https://doi.org/10.5281/zenodo.7998119
	Egypt	https://doi.org/10.5281/zenodo.7998092
	Equatorial Guinea	https://doi.org/10.5281/zenodo.7998073
	Eritrea	https://doi.org/10.5281/zenodo.7998054
	Eswatini	https://doi.org/10.5281/zenodo.7998033
	Ethiopia	https://doi.org/10.5281/zenodo.6539183
	Gabon	https://doi.org/10.5281/zenodo.7997994
	Gambia	https://doi.org/10.5281/zenodo.7997979
	Ghana	https://doi.org/10.5281/zenodo.6539195
	Guinea	https://doi.org/10.5281/zenodo.7997959
	Guinea-Bissau	https://doi.org/10.5281/zenodo.7997859
	Kenya	https://doi.org/10.5281/zenodo.6539210
	Lesotho	https://doi.org/10.5281/zenodo.7997828
	Liberia	https://doi.org/10.5281/zenodo.7997745
	Libya	https://doi.org/10.5281/zenodo.7997715
	Malawi	https://doi.org/10.5281/zenodo.7997674
	Mali	https://doi.org/10.5281/zenodo.7997657
	Mauritania	https://doi.org/10.5281/zenodo.7997601
	Morocco	https://doi.org/10.5281/zenodo.7997567
	Mozambique	https://doi.org/10.5281/zenodo.6539958
	Namibia	https://doi.org/10.5281/zenodo.7998186
	Niger	https://doi.org/10.5281/zenodo.7998205
	Nigeria	https://doi.org/10.5281/zenodo.6539979
	Rwanda	https://doi.org/10.5281/zenodo.7998279
	Senegal	https://doi.org/10.5281/zenodo.6540008
	Sierra Leone	https://doi.org/10.5281/zenodo.7998367
	Somalia	https://doi.org/10.5281/zenodo.7998431
	South Africa	https://doi.org/10.5281/zenodo.6540022
	South Sudan	https://doi.org/10.5281/zenodo.7998446
	Sudan	https://doi.org/10.5281/zenodo.7998468
	Togo	https://doi.org/10.5281/zenodo.7998501
	Tunisia	https://doi.org/10.5281/zenodo.7998519
	Uganda	https://doi.org/10.5281/zenodo.7998535
	United Republic of Tanzania	https://doi.org/10.5281/zenodo.6540040
	Zambia	https://doi.org/10.5281/zenodo.6540071
	Zimbabwe	https://doi.org/10.5281/zenodo.6540080
Asia	Cambodia	https://doi.org/10.5281/zenodo.6539134
	Indonesia	https://doi.org/10.5281/zenodo.6539208
	Lao People's Democratic Republic	https://doi.org/10.5281/zenodo.6539218
	Malaysia	https://doi.org/10.5281/zenodo.6539954
	Myanmar	https://doi.org/10.5281/zenodo.6539968
	Philippines	https://doi.org/10.5281/zenodo.6539991
	Republic of Korea	https://doi.org/10.5281/zenodo.6540000
	Taiwan Province of China	https://doi.org/10.5281/zenodo.6540029
	Thailand	https://doi.org/10.5281/zenodo.6540050
	Viet Nam	https://doi.org/10.5281/zenodo.6540059
South America	Brazil	https://doi.org/10.5281/zenodo.7998827
	Colombia	https://doi.org/10.5281/zenodo.7998839

## Data Availability

ZenodoTransport Starter Data Kit: Historical socio-transport data for selected countries in Africa, Asia, and South America (Reference data).

## Appendix

Table 5 lists the links for the country-specific Transport Starter Data Kit, which includes the dataset and data note.
